# A solitary pulmonary nodule caused by *Mycobacterium tuberculosis* var. BCG after intravesical BCG treatment: a case report

**DOI:** 10.1186/s12890-021-01475-w

**Published:** 2021-04-07

**Authors:** Mariko Itai, Mari Yamasue, Shuichi Takikawa, Kosaku Komiya, Yukiko Takeno, Yuriko Igarashi, Yasushi Takeshita, Kazufumi Hiramatsu, Satoshi Mitarai, Jun-ichi Kadota

**Affiliations:** 1 Department of Internal Medicine, National Hospital Organization Nishi-Beppu Hospital, 4548 Tsurumi, Beppu, Oita 874-0840 Japan; 2 Department of Respiratory Medicine and Infectious Diseases, Faculty of Medicine, Oita University, 1-1 Idaigaoka, Hasama-machi, Yufu, Oita 879-5593 Japan; 3 Department of Mycobacterium Reference and Research, the Research Institute of Tuberculosis, Japan Anti-Tuberculosis Association, 3-1-24, Matsuyama, Kiyose, Tokyo 204-8533 Japan; 4 Department of Internal Medicine, Tsukumi Chuo Hospital, 6011 Chinu, Tsukumi, Tsukumi, Oita 879-2401 Japan

**Keywords:** Case report, BCG immunotherapy, Pulmonary nodule, *Mycobacterium tuberculosis* var. BCG, Pulmonary tuberculosis

## Abstract

**Background:**

Intravesical instillation of bacillus Calmette–Guérin (BCG) as a treatment for superficial bladder cancer rarely causes pulmonary complications. While published cases have been pathologically characterized by multiple granulomatous lesions due to disseminated infection, no case presenting as a solitary pulmonary nodule has been reported.

**Case presentation:**

A man in his 70 s was treated with intravesical BCG for early-stage bladder cancer. After 1 year, he complained of productive cough with a solitary pulmonary nodule at the left lower lobe of his lung being detected upon chest radiography. His sputum culture result came back positive, with conventional polymerase chain reaction (PCR) identifying *Mycobacterium tuberculosis* complex. However, tuberculosis antigen-specific interferon-gamma release assay came back negative. Considering a history of intravesical BCG treatment, multiplex PCR was conducted, revealing the strain to be *Mycobacterium tuberculosis* var. BCG. The patient was then treated with isoniazid, ethambutol, levofloxacin, and para-aminosalicylic acid following an antibiotic susceptibility test showing pyrazinamide resistance, after which the size of nodule gradually decreased.

**Conclusion:**

This case highlights the rare albeit potential radiographic presentation of *Mycobacterium tuberculosis* var. BCG, showing a solitary pulmonary nodule but not multiple granulomatous lesions, after intravesical BCG treatment. Differentiating *Mycobacterium tuberculosis* var. BCG from *Mycobacterium tuberculosis* var. *tuberculosis* is crucial to determine whether intravesical BCG treatment could be continued for patients with bladder cancer.

## Background

Bacillus Calmette–Guérin (BCG) is synthesized from a weakened strain of *Mycobacterium tuberculosis* var. BCG originally developed for a tuberculosis (TB) vaccine in 1908 [1]. In 1976, Morales and coauthors published the first successful use of intravesical BCG for patients with bladder cancer [2], which has currently become the main intravesical immunotherapy for early-stage bladder cancer [3, 4]. Although BCG treatment is generally well tolerated due to its low virulence in immunocompetent patients, local and systemic complications may be observed in rare cases [5]. Indeed, studies have shown that severe adverse effects may occur in less than 5% of patients who received intravesical BCG treatment, with most of the symptoms resulting from disseminated BCG infection [6–9].

Among the severe adverse effects, lung involvement had been observed in 0.3–0.7% of patients [10–12]. While multiple granulomatous lesions due to miliary infection or interstitial lung diseases caused by hyperimmune responses have been previously documented as radiological features [3, 10], no other radiological patterns have been reported. We herein present the first case diagnosed with *Mycobacterium tuberculosis* var. BCG infection who presented with a solitary pulmonary nodule after intravesical BCG treatment for superficial bladder cancer.

## Case presentation

**Fig. 1:**
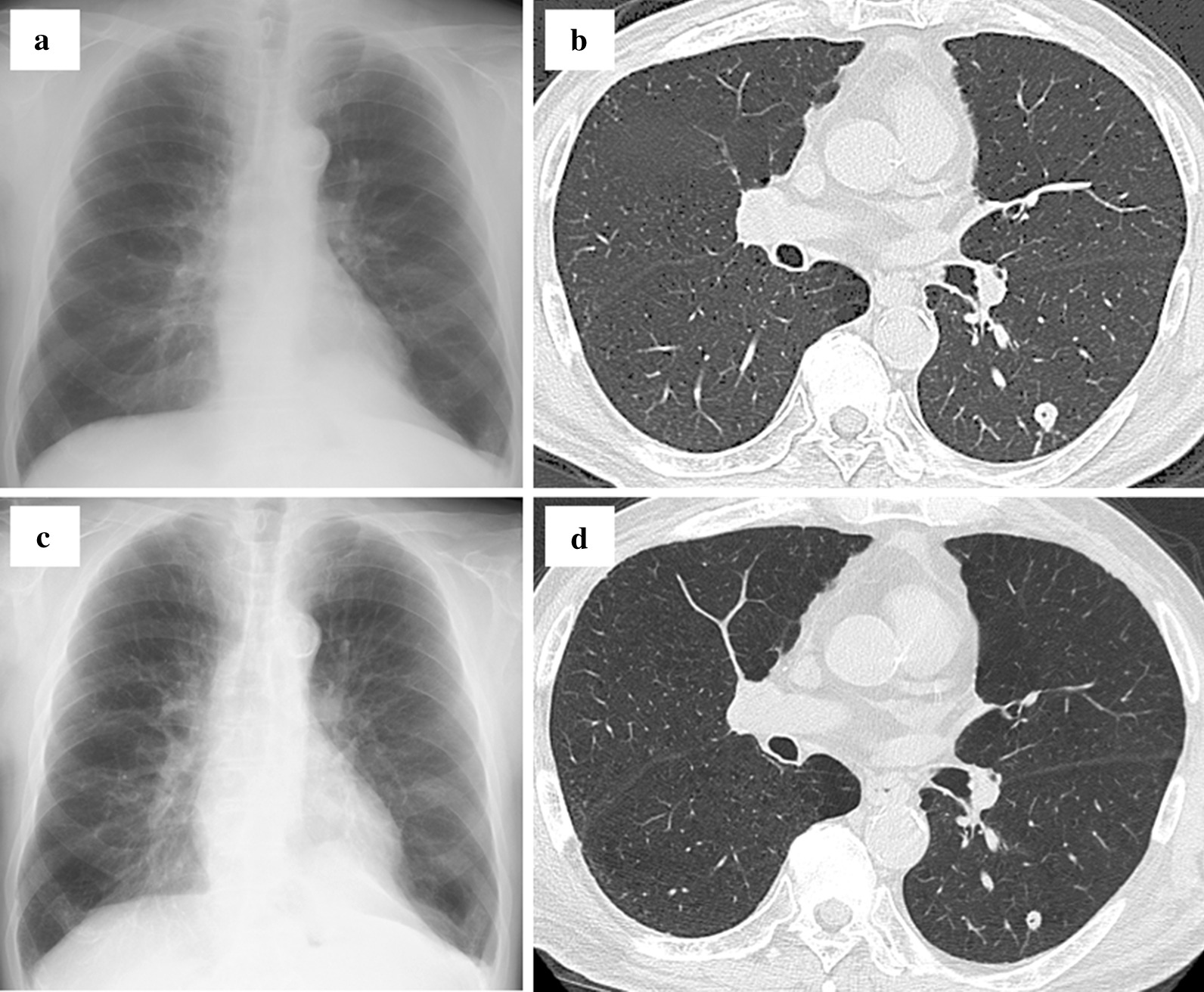
Chest X-ray and computed tomography (CT). **a** Chest X-ray on admission showing a solitary nodule in the left middle lung field. **b** Chest CT on admission showing a 12-mm nodule with a cavitary lesion and a centrilobular branching structure (tree-in-bud appearance) in the lower lobe (S^6^) of the left lung. **c** Chest X-ray 9 months after treatment initiation showing decreased size of the solitary nodule in the left middle lung field. **d** Chest CT 9 months after treatment initiation showing a XX-mm nodule diminishing the tree-in-bud appearance

A man in his 70 s with chronic obstructive pulmonary disease had undergone transurethral resection for early-stage bladder cancer, after which he received monthly intravesical BCG of 80 mg treatments for 8 times [13]. After 1 year, he started to complain of productive cough, with physical examination revealing a body temperature of 36.8 °C, an SpO_2_ of 96%, a blood pressure of 126/63 mmHg, and a heart rate of 103 beats/min. Laboratory tests revealed a normal leukocyte count (7400 cells/μL) and slightly elevated serum levels of C-reactive protein (0.03 mg/dL), while chest X-ray detected a solitary pulmonary nodule in left middle lung field. Chest computed tomography showed a solitary nodule with a cavitary lesion in left lower lobe of the lung (segment 6) as shown in Fig. 1a and b. A tree-in-bud appearance characterized by a centrilobular branching structure was also found near the nodule.

Although his sputum acid-fast bacilli smear came back negative, liquid culture (Mycobacteria Growth Indicator Tube, Becton Dickinson, Sparks, MD) revealed a positive result, with conventional PCR identifying *Mycobacterium tuberculosis* complex. Thereafter, he started receiving multiple anti-TB drugs, including isoniazid, rifampicin, ethambutol, and pyrazinamide as treatment for *Mycobacterium tuberculosis* var. *tuberculosis* infection. Two weeks later, rifampicin was replaced by levofloxacin due to fever and fatigue suspected to have been adverse effects of rifampicin.

Interestingly, we had a negative result for the TB-specific interferon-gamma release assay performed as an auxiliary diagnosis for active *Mycobacterium tuberculosis* infection. Considering a history of intravesical BCG treatment for bladder cancer, multiplex PCR was conducted, subsequently revealing the strain to be *Mycobacterium tuberculosis* var. BCG [14]. Urinary acid-fast bacilli culture was confirmed to be negative. Given that routine antibiotic susceptibility test, the Kyokuto PZA test commercially available in Japan showed pyrazinamide resistance [15], the patient was started on ethionamide instead of pyrazinamide. Six weeks later, we observed liver injury, which was suspected to be induced by ethionamide because other potential causes including viral and alcoholic influence were excluded. Liver images using ultrasound and CT did not found abnormality, and we did not conduct liver biopsy. Consequently, ethionamide was replaced by para-aminosalicylic acid and then liver enzymes returned to normal range. Nine months after the treatment initiation, the size of the nodule significantly decreased, diminishing the surrounding tree-in-bud appearance, as shown in Fig. 1c and d. The treatment was planned to continue up to 12 months according to some previous reports [8], despite no official treatment guideline for *Mycobacterium tuberculosis* var. BCG.

**Table 1:** Review of the previous cases of pulmonary complication for intravesical BCG treatment

Total cases	n = 39
Male (sex was not described in 5 cases)	34 (97)
Age, year	68 (64–75)
Smoker	7 (18)
Diabetes mellitus	3 (8)
Alcohol abuse	1 (3)
Malignancy	3 (8)
HIV infection	1 (3)
COPD	4 (10)
Asthma	1 (3)
Interstitial pneumonia	1 (3)
Old pulmonary tuberculosis	4 (10)
Ischemic heart disease	4 (10)
BPH	3 (8)

Bladder cancer treatment	
Only BCG therapy	21 (54)
BCG + Mitomycin C	1 (3)
TURBT + BCG (± Mitomycin C)	15 (38)
Cystectomy (± TURBT) + BCG	2 (5)
BCG therapy before onset, times	6 (5–9)
Time from final BCG injection to onset, day	30 (7–154)

Chest CT findings	
Miliary pattern (diffused well-defined small nodular opacity)	22 (56)
Diffused ill-defined small nodular opacity	3 (8)
Multiple nodules	1 (3)
Grand glass opacity	7 (18)
Consolidation	9 (23)
Interlobular septae thickening	1 (3)
Bronchial wall thickening	1 (3)
Pleural effusion	5 (13)
Mediastinal and/or hilar lymphadenopathy	3 (8)

Data are presented as the number (%) or median Interquartile range

*HIV* human immunodeficiency virus, *COPD* chronic obstructive pulmonary disease, *BPH* benign prostatic hyperplasia, *BCG* intravesical Bacillus Calmette–Guérin treatment, *TURBT* transurethral resection of the bladder tumor

## Discussion and conclusion

We herein present a case of *Mycobacterium tuberculosis* var. BCG infection showing a solitary pulmonary nodule after intravesical BCG treatments for superficial bladder cancer. Pulmonary complications due to intravesical BCG treatment are quite rare. We searched cases of *Mycobacterium tuberculosis* var. BCG infection using the search strategy (bladder cancer) AND [(BCG) OR (mycobacterium bovis)] AND [(lung) OR (pulmonary)] through the PubMed database assessed October 29, 2020 and summarized its clinical characteristics in Table 1 [9, 16–46]. Accordingly, most of the cases had multiple granulomatous lesions referred to as disseminated infection, with no report presenting a solitary pulmonary nodule.

Our case presented with a solitary pulmonary nodule without any other involvement. Although a tree-in-bud appearance, perhaps due to the inhalation of bacteria, was observed near the nodule, the patient had no history of being near cows carrying *Mycobacterium tuberculosis* var. BCG or BCG powder inhalation. Thus, the tree-in-bud appearance may probably be attributed to the spread of bacteria from the nodule. Although *Mycobacterium tuberculosis* var. BCG in the nodule can be suspected to have been transferred from the bladder through the bloodstream, it still remains uncertain why only a solitary nodule, instead of multiple granulomatous lesions, had formed. While an immunocompromised condition can be considered a risk factor for disseminated *Mycobacterium tuberculosis var.* BCG infection [8], the patient in the current case had no underlying diseases apart from bladder cancer, which may perhaps have influenced the formation of the solitary nodule. However, even in immunocompetent hosts disseminated pattern has been observed. We are unable to provide clear reasons for the solitary formation in this case.

The purpose of distinguishing *Mycobacterium tuberculosis* var. BCG from *Mycobacterium tuberculosis* var. *tuberculosis* in clinical practice warrants discussion. In Japan, TB-specific interferon-gamma release assay is allowed to test as an auxiliary diagnosis for active *Mycobacterium tuberculosis* infection by universal health insurance coverage. TB-specific interferon-gamma release assay results came back to negative in this case, which became a clue to suspect of *Mycobacterium tuberculosis* var*.* BCG infection. However, because sensitivity of the test is reported approximately 80% [47], it should be noted that *Mycobacterium tuberculosis* var*.* BCG cannot be suspected by the negative TB-specific interferon-gamma release assay results. While treatment regimens for *Mycobacterium tuberculosis* var*.* BCG closely resemble those for *Mycobacterium tuberculosis*, *Mycobacterium tuberculosis* var. BCG is naturally resistant to pyrazinamide [48]. As far as drug susceptibility tests are concerned, discriminating between both strains in general practice may not always be required. Nevertheless, if *Mycobacterium tuberculosis* var. BCG infection is diagnosed after intravesical BCG treatment, the aforementioned immunotherapy would no longer be appropriate. As such, even when a solitary pulmonary nodule is found among patients receiving intravesical BCG treatment for superficial bladder cancer, ruling out *Mycobacterium tuberculosis* var. BCG infection is required.

## Data Availability

Data are available in this manuscript.

## Abbreviations

BCG: Bacillus Calmette–Guérin
CT: Computed tomography
PCR: Polymerase chain reaction
